# Prevalence of gingival recession after orthodontic treatment of infraversion and open bite

**DOI:** 10.1007/s00056-018-0159-8

**Published:** 2018-09-21

**Authors:** Juan-Juan Ji, Xu-Dong Li, Qun Fan, Xiao-Jun Liu, Shuang Yao, Zhi Zhou, Shuang Yang, Yong Shen

**Affiliations:** 1 Department of Orthodontics, The Second People’s Hospital of Yunnan, No. 176 Qingnian Road, 650021 Kunming, Yunnan China; 2 Department of Prosthodontics, The Affiliated Stomatology Hospital of Kunming Medical Collage, 650031 Kunming, Yunnan China

**Keywords:** Open bite, Infraversion, Occlusion, Periodontal tissue, Gingival recession, Offener Biss, Infraposition, Okklusion, Parodontium, Gingivarezession

## Abstract

**Purpose:**

Aim of the present study was to investigate the prevalence of gingival recession and related factors in teeth with low occlusal function (open bite and infraversion) after orthodontic treatment.

**Methods:**

From January 2014 to December 2017, 403 patients received orthodontic treatment. Their gingival recession and related factors before and after treatment were retrospectively analyzed.

**Results:**

The prevalence of gingival recession in patients with infraversion and open bite after orthodontic treatment were 80.6 and 75.0%, respectively; these values were 43.4 and 47.5% before treatment, respectively. Notably, the Miller index of gingival recession increased after orthodontic treatment (*P* < 0.05). The risk of gingival recession in patients with infraversion or open bite after orthodontic treatment was remarkably higher than the risk in other patients (odds ratio [OR] = 16.712 and 5.073, respectively); the gingival recession rate was related to treatment with tooth extraction (OR = 2.043), as well as gingival biotype (OR = 0.341) and gingival index (GI) before orthodontic treatment (OR = 97.404; *P* < 0.05).

**Conclusions:**

Patients with these two types of low occlusal function are more likely to exhibit gingival recession after orthodontic treatment. Moreover, the prevalence of gingival recession after orthodontic treatment is higher among patients who have undergone tooth extraction during orthodontic treatment, and among those who exhibit thin gingival biotype and high gingival index before orthodontic treatment.

## Zusammenfassung

**Ziel:**

Ziel der vorliegenden Studie war die Untersuchung der Prävalenz von Gingivarezessionen und verwandter Faktoren bei Zähnen mit geringer okklusaler Funktion (offener Biss und Infraposition) nach kieferorthopädischer Behandlung.

**Methoden:**

Von Januar 2014 bis Dezember 2017 wurden 403 Patienten kieferorthopädisch behandelt. Gingivale Rezessionen und damit verbundene Faktoren wurden vor und nach der Behandlung retrospektiv analysiert.

**Ergebnisse:**

Die Prävalenz der Gingivarezession bei Patienten mit Infraposition und offenem Biss nach kieferorthopädischer Behandlung lag bei 80,6 bzw. 75,0 %; die entsprechenden Werte vor der Behandlung betrugen 43,4 bzw. 47,5 %. Insbesondere der Miller-Index der Gingivarezession stieg nach kieferorthopädischer Behandlung (*p* < 0,05). Das Risiko einer Gingivarezession war bei Patienten mit Infraposition oder offenem Biss nach kieferorthopädischer Behandlung deutlich höher als bei anderen Patienten (OR [Odds Ratio] 16,712 bzw. 5,073). Die Gingivarezessionsrate war assoziiert mit Zahnextraktion (OR 2,043), dem Gingivabiotyp (OR 0,341) und dem Gingivaindex (GI) vor kieferorthopädischer Behandlung (OR 97,404, *p* < 0,05).

**Schlussfolgerungen:**

Patienten mit offenem Biss und Zähnen in Infraposition zeigen nach einer kieferorthopädischen Behandlung eher Gingivarezessionen. Außerdem ist die Prävalenz der Gingivarezession nach kieferorthopädischer Behandlung höher bei Patienten, die sich während der kieferorthopädischen Behandlung einer Zahnextraktion unterzogen haben, und bei Patienten, die einen dünnen Gingivabiotyp und einen hohen Gingivaindex vor der kieferorthopädischen Behandlung aufwiesen.

## Introduction

Suitable occlusive stimulation can affect the structure of periodontal tissue, as well as maintaining and reshaping periodontal tissue, playing a key role in the regulation of teeth and periodontal stability. The lack of appropriate occlusal stimulation disrupts the balance of the masticatory system and causes a series of changes, such as root resorption and periodontal ligament weakness [1–3]. As part of the periodontal tissue, the gingiva plays an important role in maintaining the appearance of the teeth and protecting the tooth surface. Contraction of the gingiva increases the space between teeth, sensitivity of teeth, susceptibility to cervical caries, and other adverse consequences [4–6]. It has been reported that the prevalence of gingival recession in patients with teeth that exhibit low occlusal function is higher than that in patients that exhibit normal occlusion, due to the lack of appropriate occlusal stimulation.

A method to modify low occlusal function is orthodontic treatment, which can relieve congestion, align the dentition, and establish a good occlusal relationship. The relevant principle is to move the teeth by alveolar bone reconstruction with external force [7–9].

However, the relationship between orthodontic treatment and gingival recession has been controversially discussed in clinical research. Studies have shown that, in patients who exhibit gingival recession caused by occlusal trauma, the alveolar bone is remodeled and the condition of gingival recession is improved by orthodontic treatment [10]. It has also been reported that the prevalence of gingival recession is increased by orthodontic treatment in patients with mandibular anterior teeth anteversion and in patients requiring upper dental arch expansion. However, this improvement is not statistically different from that of the untreated group [11].

Infraversion, open bite, and missing teeth are very common types of malocclusion, which cause weakened bite force and lead to a state of low occlusal function. The effect of orthodontic treatment on the gingival recession of teeth with poor occlusion has not yet been reported. Notably, there have been no reports regarding the tendency to reduce or aggravate gingival recession of the teeth due to poor occlusion. A retrospective study was conducted to explore the effects of poor occlusion on gingival recession and its relative factors by analyzing the prevalence of gingival recession after orthodontic treatment in patients with infraversion and open bite; this is expected to provide a basis for a safer clinical pathway of orthodontic treatment for teeth with low occlusal function.

## Materials and methods

### Subjects and indicators

From January 2014 to December 2017, 403 patients underwent complete orthodontic treatment. Inclusion criteria were the following: (1) patients without systemic diseases; (2) patients 11–43 years of age; (3) patients without a long history of medication; (4) nonpregnant women; (5) patients requiring orthodontic treatment; (6) patients who provided informed consent for orthodontic treatment and participation in the study, and who began and finished the entire treatment during the selected period; (7) patients without occlusal trauma; (8) patients diagnosed with mild or moderate chronic periodontitis and gingival recession level 2 by a periodontal specialist; (9) patients whose gingiva was at the inflammatory resting stage, at 3–6 months after basic periodontal treatment, whose bleeding index was less than 25% in full mouth probing examination, and who exhibited good oral hygiene and compliance. The study models, photographs, medical records, and panoramic radiographs of the selected patients before treatment were carefully reviewed.

## Data collection

General data, such as case number, sex, age, and smoking status, were collected. The clinical examination included the following: judgment of the presence of infraversion, open bite (not including infraversion), missing teeth, and/or occlusal trauma; gingival biotype, vertical facial forms (high, even, and low angle), gingival index (GI) before and after orthodontic treatment, periodontal index (PI), and plaque index. Tooth extraction was also recorded as part of the treatment plan. According to the Miller gingival recession classification [3], gingival recession was classified: Class I, gingival recession does not exceed the mucogingival junction, no loss of bone or soft tissue is observed in adjacent tooth space; Class II, gingival recession exceeds the mucogingival junction, no loss of bone or soft tissue is observed in adjacent tooth space; Class III, gingival recession does not exceed the mucogingival junction, and there is a loss of periodontal tissue in adjacent teeth; Class IV, gingival recession exceeds the mucogingival junction, and there is a loss of periodontal tissue in adjacent teeth. We used probing transparency in the sulcus [12] to determine gingival biotype.

## Statistical analysis

The SPSS17.0 statistical software package was used for data processing. The statistical description was expressed as absolute number or rate. Paired rank data were evaluated by using the Wilcoxon rank sum test; logistic regression modeling was used to identify risk factors for gingival recession by univariate and multivariate logistic regression. All covariates with *P*-values less than 0.05 were included in the multivariate model. All statistical analyses were performed by SPSS17.0, with *P*-values <0.05 considered significant.

## Results

### Factors influencing gingival recession

A total of 403 orthodontic patients were recruited, including 118 men (29.3%) and 285 women (70.7%); 222 patients were younger than 18 years of age (55.1%) and 181 were older than 18 years of age (44.9%). There were 20 patients with active smoking habits (5%) and 383 patients who were nonsmokers (95%). The oral conditions of the subjects are shown in Table 1.

**Table 1:** Results from logistic regression of gingival recession (*n* = 403)

Variable	*n* tested	Gingival recession		Univariate analysis		Multivariate analysis	
		*n*	% (95% CI)	Odds ratio (95% CI)	*p* value	Odds ratio (95% CI)	*p* value
Infraversion							
No	228 (56.6)	94	41.2 (34.8–47.6)	^a^	–	–	–
Yes	175 (43.4)	141	80.6 (74.7–86.5)	5.912 (3.739–9.346)	<0.001	16.712 (7.896–35.373)	<0.001
Open bite							
No	323 (80.1)	175	54.2 (48.8–59.6)	^a^	–	–	–
Yes	80 (19.9)	60	75.0 (65.5–84.5)	2.537 (1.462–4.404)	0.001	5.073 (2.107–12.213)	<0.001
Gender							
Male	118 (29.3)	61	51.7 (42.7–60.7)	^a^	–	–	–
Female	285 (70.7)	174	61.1 (55.4–66.8)	1.465 (0.950–2.257)	0.084	–	–
Age, years							
<18	222 (55.1)	114	51.4 (44.8–58.0)	0.523 (0.349–0.786)	0.002	–	–
≥18	181 (44.9)	121	66.9 (60.0–73.8)	^a^	–	–	–
Smoking							
No	383 (95.0)	224	58.5 (36.9–80.1)	^a^	–	–	–
Yes	20 (5.0)	11	55.0 (50.0–60.0)	0.868 (0.351–2.143)	0.758	–	–
Missing teeth							
No	373 (92.6)	218	58.4 (53.4–63.4)	^a^	–	–	–
Yes	30 (7.4)	17	56.7 (39.0–74.4)	0.930 (0.439–1.970)	0.849	–	–
Tooth trauma							
No	257 (63.8)	154	59.9 (53.9–65.9)	^a^	–	–	–
Yes	146 (36.2)	81	55.5 (47.4–63.6)	0.833 (0.553–1.257)	0.385	–	–
Facial forms							
Even angle	171 (42.4)	90	52.6 (45.1–60.1)	^a^	–	–	–
Low angle	105 (26.1)	60	57.1 (47.6–66.6)	1.200 (0.736–1.958)	0.465	–	–
High angle	127 (31.5)	85	66.9 (58.7–75.1)	1.821 (1.131–2.932)	0.014	–	–
Tooth extraction							
No	223 (55.3)	105	47.1 (40.5–53.7)	^a^	–	–	–
Yes	180 (44.7)	130	72.2 (65.7–78.7)	2.922 (1.922–4.442)	<0.001	2.043 (1.041–4.011)	0.038
Gingival biotype							
Thin	151 (37.5)	110	72.8 (65.7–79.9)	^a^	–	–	–
Thick	252 (62.5)	125	49.6 (43.4–55.8)	0.367 (0.237–0.567)	<0.001	0.341 (0.159–0.730)	0.006
Gingival index before orthodontic treatment							
0	251 (62.3)	88	35.1 (29.2–41.0)	^a^	–	–	–
1	129 (32.0)	124	96.1 (92.8–99.4)	45.936 (18.109–116.526)	<0.001	–	–
2	19 (4.7)	19	100	–	–	–	–
3	4 (1.0)	4	100	–	–	97.404 (31.128–304.789)	<0.001
Changes of periodontal index							
Reduced	147 (36.5)	86	58.5 (50.5–66.5)	–	–	–	–
No change	256 (63.5)	149	58.2 (52.2–64.2)	0.988 (0.655–1.491)	0.953	–	–
Changes of plaque index							
Reduced	185 (45.9)	114	61.6 (54.6–68.6)	–	–	–	–
No change	217 (53.9)	121	55.8 (49.2–62.4)	–	–	–	–
Increased	1 (0.2)	0	0	0.779 (0.549–1.105)	0.162	–	–
Changes of gingival index							
Reduced	175 (43.5)	107	61.1 (53.9–68.3)	–	–	–	–
No change	227 (56.3)	128	56.4 (49.9–62.9)	–	–	–	–
Increased	1 (0.2)	0	0	0.834 (0.583–1.191)	0.834	–	–

*CI* confidence interval

^a^Reference group

### Gingival recession

Before orthodontic treatment, the prevalences of gingival recession in patients with infraversion, open bite, and other groups were 43.4%, 47.5%, and 26.3%, respectively. After orthodontic treatment, the respective prevalences of gingival recession were 80.6, 75, and 29.3%. When the Miller index was used to evaluate the severity of gingival recession of each tooth before and after orthodontic treatment, the severity of gingival recession was the highest in infraversion, followed by open bite, and then normal teeth before orthodontic treatment; the trend was similar after orthodontic treatment. The gingival recession of both infraversion and open bite was elevated by orthodontics (Z = −13.49, *P* < 0.001; Z = −18.63, *P* < 0.001), but the increased degree of recession was not different between them (Z = −1.186, *P* = 0.236; Table 2).

**Table 2:** Comparisons of Miller index of gingival recession before and after orthodontic treatment

Category	*N*	Miller index of gingival recession before orthodontic treatment			Miller index of gingival recession after orthodontic treatment			Variation	*Z*	*P*
		0, *n* (%)	1, *n* (%)	2, *n* (%)	0, *n* (%)	1, *n* (%)	2, *n* (%)			
Infraversion	424	237 (55.9)	182 (42.9)	5 (1.2)	65 (15.3)	344 (81.1)	15 (3.5)	0.42925	−13.49	<0.001
Open bite	746	597 (80.0)	149 (20.0)	0	267 (35.8)	462 (61.9)	17 (2.3)	0.46515	−18.63	<0.001
Others	9322	9028 (96.8)	294 (3.2)	0	9025 (96.8)	297 (3.2)	0	0.00032	−1.732	0.083

### Univariate and multivariate logistic regression analysis

Univariate and multivariate logistic regression analysis was performed by using gingival recession after orthodontic treatment as the dependent variable (0 for without, 1 for with); the existence of open bite and infraversion, as well as gender, age, smoking, missing teeth, tooth trauma, facial forms, tooth extraction, gingival biotype, gingival index before orthodontic treatment, and periodontal index after orthodontic treatment, plaque index, and changes of gingival index were independent variables. After controlling for the influence of other factors on gingival recession, patients with open bite or infraversion had significantly higher prevalences of gingival recession after orthodontic treatment than patients without open bite or infraversion. Moreover, tooth extraction was a risk factor for gingival recession. Patients with thin gingival biotype and high gingival index before orthodontic treatment had higher risk of gingival recession (Table 1).

## Discussion

It is well known that external force can cause remodeling of periodontal tissue. Frequent occlusal trauma increases the strength of occlusal force and often leads to resorption of alveolar bone. When occlusal trauma is removed, normal bite force is restored and alveolar bone is rebuilt with increased bone volume. However, in addition to the increased occlusal force, abnormal occlusal force also includes reduced occlusal force, known as low occlusal function. Due to the loss of proper stimulation from occlusal force, the periodontal tissue is recessively reacted through root resorption and alveolar bone absorption. Clinically, infraversion is the most common example of low occlusal function, followed by teeth without occlusal contact except infraversion, such as open bite and missing teeth in the opposite jaw, and other malocclusive teeth. The first two situations are easily diagnosed and recorded; thus, we examined infraversion and open bite as representatives of teeth with low occlusal function in this retrospective study. In daily clinical orthodontic work, we found that teeth with low occlusal function are more prone to gingival recession after orthodontic treatment. Therefore, we assumed that teeth with infraversion and open bite are more prone to gingival recession than teeth with normal occlusion.

Our results showed that the prevalences of gingival recession after orthodontic treatment were higher in patients with infraversion and open bite. We also compared changes of gingival index before and after orthodontic treatment. Gingival index, revised by Silness and Löe in 1967 [13], is used to evaluate the severity of gingivitis on a scale of 0–4; a higher number represents more severe gingivitis. The results in this study suggested that more severe gingivitis was associated with increased inflammatory reaction to orthodontic treatment, playing a role in support of periodontal tissue and leading to gingival recession; however, its mechanism remains unclear.

The overall results suggested that teeth with low occlusal function are more likely to exhibit gingival recession, which is consistent with observed phenomena in clinical practice and our previous assumptions. Notably, this may be related to gingival disuse caused by low occlusal function.

By moving the location of teeth, orthodontic treatment can restore a normal occlusal relationship. Therefore, it is an effective method to treat teeth with low occlusal function. However, the effect of orthodontic treatment on gingival atrophy is controversial. Some scholars have found that correct orthodontic treatment can help to improve gingival recession, whereas other scholars believe that orthodontic treatment can cause or aggravate gingival recession [14, 15]. These studies have not involved teeth with low occlusal function; thus, the effect of orthodontic treatment on gingival status in patients with low occlusal function is unclear. This study found that the gingival recession rates of infraversion and open bite after orthodontic treatment were higher than those before treatment. Indeed, teeth with these two types of low occlusal function are more susceptible to gingival recession before orthodontic treatment. The orthodontic force is a cause for remodeling within the periodontal tissue, which may induce the pathological mechanism of gingival recession when the gingiva is in a subhealthy state. This may explain the aggravation of gingival recession in cases of infraversion or open bite induced by orthodontic treatment.

In addition, many studies regarding changes in hard tissues, such as alveolar bone and root, caused by occlusal function changes have been reported; further, many related factors have been studied. However, changes in gingival tissue are less well described [16–18]. Questions remain regarding the factors associated with an increase of gingival recession rate after orthodontic treatment. Our results revealed that after adjusting for other factors, the prevalence of gingival recession in patients with infraversion or open bite was higher than in patient without infraversion and open bite after orthodontic treatment; this may be associated with the sudden stress to gingiva that have experienced long-term disuse due to low occlusal function. However, the specific mechanism needs to be elucidated by further experimental studies. Patients with tooth extraction are more likely to exhibit gingival recession than those without, which is probably due to the greater range of tooth movement in treatment with tooth extraction than in treatment without tooth extraction. Different gingival biotypes could lead to different prognoses. Seibert and Lindhe [19] divided the gingiva into two types, thin and thick, according to different gingival margin, keratinized gingiva, crown shape, and contact point of adjacent teeth; we propose the concept of “thin fan-shaped gingiva” and “thick platform gingiva.” Gingival recession in orthodontic treatment occurs less frequently in patients with thick gingival type, compared with patients with thin gingival type, consistent with the results of Cook et al. [20]. Khorramdel et al. [21] compared the bone thickness of the labial side of upper anterior teeth between patients with thin gingiva and those with thick gingiva by using cone beam computed tomography (CBCT) and proved that the periodontal biotype is significantly correlated with the thickness of labial bone. Fu et al. [22] found that there was a correlation between the thickness of the gingiva and the bone thickness at the labial and palatal side in cadaveric head specimens. Thus, the bone mass of the alveolar bone corresponding to the thick gingival biotype is greater than that corresponding to the thin gingival biotype; there is increased periodontal supporting tissue, compared with the thin gingival biotype. Therefore, the prevalence of bone resorption and bone fenestration, as well as the prevalence of gingival recession, is low when the thick gingival type is subjected to external force.

## Conclusion

This study found that patients with infraversion and open bite were more susceptible to gingival recession before orthodontic treatment, and that orthodontic treatment aggravated the prevalence of gingival recession. Patients who underwent tooth extraction treatment, with thin gingival biotype and high gingival index before orthodontic treatment, had a higher prevalence of gingival recession after orthodontic treatment. Due to the remodeling mechanism of orthodontic force on teeth with low occlusal function, safeguards for such patients during orthodontic treatment must be further studied.
